# Digitally Supported Lifestyle Intervention to Prevent Type 2 Diabetes Through Healthy Habits: Secondary Analysis of Long-Term User Engagement Trajectories in a Randomized Controlled Trial

**DOI:** 10.2196/31530

**Published:** 2022-02-24

**Authors:** Piia Lavikainen, Elina Mattila, Pilvikki Absetz, Marja Harjumaa, Jaana Lindström, Elina Järvelä-Reijonen, Kirsikka Aittola, Reija Männikkö, Tanja Tilles-Tirkkonen, Niina Lintu, Timo Lakka, Mark van Gils, Jussi Pihlajamäki, Janne Martikainen

**Affiliations:** 1 School of Pharmacy University of Eastern Finland Kuopio Finland; 2 VTT Technical Research Centre of Finland Ltd Espoo Finland; 3 School of Medicine Institute of Public Health and Clinical Nutrition University of Eastern Finland Kuopio Finland; 4 Faculty of Social Sciences Tampere University Tampere Finland; 5 Department of Public Health and Welfare Finnish Institute for Health and Welfare Helsinki Finland; 6 Endocrinology and Clinical Nutrition Department of Medicine Kuopio University Hospital Kuopio Finland; 7 Institute of Biomedicine University of Eastern Finland Kuopio Finland; 8 Department of Clinical Physiology and Nuclear Medicine Kuopio University Hospital Kuopio Finland; 9 Foundation for Research in Health Exercise and Nutrition Kuopio Research Institute of Exercise Medicine Kuopio Finland

**Keywords:** type 2 diabetes, user engagement, digital behavior change intervention, trajectories, habit formation, mobile health

## Abstract

**Background:**

Digital health interventions may offer a scalable way to prevent type 2 diabetes (T2D) with minimal burden on health care systems by providing early support for healthy behaviors among adults at increased risk for T2D. However, ensuring continued engagement with digital solutions is a challenge impacting the expected effectiveness.

**Objective:**

We aimed to investigate the longitudinal usage patterns of a digital healthy habit formation intervention, BitHabit, and the associations with changes in T2D risk factors.

**Methods:**

This is a secondary analysis of the StopDia (Stop Diabetes) study, an unblinded parallel 1-year randomized controlled trial evaluating the effectiveness of the BitHabit app alone or together with face-to-face group coaching in comparison with routine care in Finland in 2017-2019 among community-dwelling adults (aged 18 to 74 years) at an increased risk of T2D. We used longitudinal data on usage from 1926 participants randomized to the digital intervention arms. Latent class growth models were applied to identify user engagement trajectories with the app during the study. Predictors for trajectory membership were examined with multinomial logistic regression models. Analysis of covariance was used to investigate the association between trajectories and 12-month changes in T2D risk factors.

**Results:**

More than half (1022/1926, 53.1%) of the participants continued to use the app throughout the 12-month intervention. The following 4 user engagement trajectories were identified: terminated usage (904/1926, 46.9%), weekly usage (731/1926, 38.0%), twice weekly usage (208/1926, 10.8%), and daily usage (83/1926, 4.3%). Active app use during the first month, higher net promoter score after the first 1 to 2 months of use, older age, and better quality of diet at baseline increased the odds of belonging to the continued usage trajectories. Compared with other trajectories, daily usage was associated with a higher increase in diet quality and a more pronounced decrease in BMI and waist circumference at 12 months.

**Conclusions:**

Distinct long-term usage trajectories of the BitHabit app were identified, and individual predictors for belonging to different trajectory groups were found. These findings highlight the need for being able to identify individuals likely to disengage from interventions early on, and could be used to inform the development of future adaptive interventions.

**Trial Registration:**

ClinicalTrials.gov NCT03156478; https://clinicaltrials.gov/ct2/show/NCT03156478

**International Registered Report Identifier (IRRID):**

RR2-10.1186/s12889-019-6574-y

## Introduction

Type 2 diabetes (T2D) is currently one of the most prevalent noncommunicable diseases burdening public health globally [1]. It is a progressing disease that compromises health-related quality of life [2,3], introduces severe comorbidities, and increases premature mortality [4,5]. It also has a significant economic impact on individuals, health systems, and societies [6]. However, research shows that T2D could be prevented efficiently by lifestyle improvement [7-9]. This highlights the need for early diabetes prevention. In addition, plausible effects of lifestyle changes are reported to sustain for several years [10,11]. Still, after 20 years of evidence of effectiveness, health care systems struggle to find and implement scalable individual-level lifestyle change support in routine practice.

Digital health interventions may offer a scalable way to prevent T2D by providing early support for healthy behavior improvement among individuals at increased risk of T2D with minimal burden to health systems. A recent review supported findings on the effectiveness of technology-driven T2D prevention interventions in producing short-term (≤6 months) and long-term (≥12 months) weight loss, with the number of behavior change techniques applied positively associated with effectiveness [12]. However, continuous user engagement in digital solutions remains a challenge [13-15], impacting the expected effectiveness. For this, finding out who will actively use and potentially benefit from digital solutions is essential for providing the right care to the right person at the right time, which, in turn, will potentially improve the personalization and optimization of the care.

The BitHabit app was developed in the Finnish Stop Diabetes (StopDia) project to provide adults at increased risk of T2D an easy way to form healthy lifestyle habits [16]. The applicability of the BitHabit app was further evaluated in the Finnish health care system in a 12-month randomized controlled trial (RCT) [16]. The aims of this study were to (1) identify long-term user engagement trajectories of the BitHabit app among the participants, (2) examine predictors of the trajectories, and (3) investigate associations between the trajectories and changes in T2D risk factors during the trial.

## Methods

### Study Design and Participants

This study is a secondary analysis of the StopDia trial, which was an unblinded parallel RCT (ClinicalTrials, NCT03156478). The participants of the trial were randomly allocated to a digital intervention group (DIGI; n=967), a group combining the digital intervention and face-to-face group coaching (DIGI+GROUP; n=971), or a control group (n=969). Randomization was done using a computerized randomization system with 1:1:1 allocation after baseline examinations. In this study, only data from the digital intervention arms (ie, DIGI and DIGI+GROUP) were used. The RCT study protocol, including the intervention lifestyle objectives and outcome measures, has been described in more detail in earlier work [16].

The participants were recruited from several communication channels, such as social media, newspapers, radio, television, websites, health care and social service units, and community pharmacies, by encouraging people to visit the website of the project to fill in the StopDia Digital Screening Tool [17]. The participants were adults aged 18 to 74 years living in 3 provinces of Finland and being at increased risk of T2D as identified with the StopDia Digital Screening Tool including the Finnish Diabetes Risk Score (FINDRISC) developed to estimate the 10-year risk of developing drug-treated T2D [18]. Adults at increased risk of T2D, who scored at least 12 points in the FINDRISC, had repeatedly measured impaired fasting glucose or glucose tolerance, or had a history of gestational diabetes, were invited to participate in the study. Other inclusion criteria were a possibility to use a computer, smartphone, or tablet with internet access; having a mobile phone number; and having adequate Finnish language skills. The exclusion criteria were current type 1 or 2 diabetes, current pregnancy or breastfeeding, having an active cancer, and being less than 6 months from active cancer treatment.

### Intervention

Participants in the DIGI and DIGI+GROUP received access to the BitHabit app for the 1-year intervention period in 2017-2019. The BitHabit app was designed in the StopDia project based on principles derived from habit formation theories [19,20] and the Self-Determination Theory [21]. The BitHabit app has been described in more detail earlier [22]. Briefly, the main goal of the app was to help the participants form lifestyle habits that would help prevent T2D. The app provided the user with a broad selection of behaviors in 13 different categories related to diet, physical activity, sleep, positive mood, stress management, smoking, and alcohol consumption. App use included browsing and selecting context-specific minimum-effort behaviors, reporting performances, and monitoring progress. Automatization of the behaviors into frequently repeated habits was facilitated by the nature of the behaviors (simple, with contextual triggers) as well as reminders for reporting performances.

In addition to using the BitHabit app, DIGI+GROUP members also participated in group coaching consisting of 6 meetings organized in local health care centers, as explained in detail previously [22].

### Study Visits

At the first examination visit, after the participants had signed informed consent, clinical measurements, including body weight and height, and waist circumference, were taken by a study nurse in a local health care center. Thereafter, participants received a link to a StopDia Digital Questionnaire with standardized and validated questions in 3 sets to assess diet quality, eating behavior, physical activity, sedentary time, smoking, quality of life, stress, sleep, and several other factors related to T2D risk. The second examination consisted of laboratory measures, including blood glucose, taken in the local health care center. All baseline assessments were repeated at the 12-month follow-up.

After using the app for 1 to 2 months, the DIGI and DIGI+GROUP members were also asked to answer questions on app user experience. They were also asked if they would recommend the BitHabit app to their friends, with possible responses ranging from 0 (not at all likely to recommend) to 10 (very likely to recommend).

### Assessments

#### Baseline Characteristics

At baseline, the participants reported on the following clinical factors: age (categorized as <50, 50-59, and ≥60 years), sex, BMI (kg/m^2^), waist circumference (cm), glycated hemoglobin A_1c_ (HbA_1c_; mmol/mol), FINDRISC, diet quality, and physical activity. Obesity was defined as BMI ≥30 kg/m^2^, and abdominal obesity was defined as a waist circumference ≥88 cm for women and ≥102 cm for men [23]. Diet quality was assessed using the Healthy Diet Index that ranges between 0 and 100, and is calculated as the sum of scores for 7 main domains, including meal pattern (0-10 points), grains (0-20 points), fruits and vegetables (0-20 points), fats (0-15 points), fish and meat (0-10 points), dairy (0-10 points), and snacks and treats (0-15 points), with higher scores indicating a healthier diet [24]. Perceived self-efficacy related to nutrition was measured with the Nutrition Emotional Barriers Self-Efficacy Score, with higher scores indicating better emotional barrier self-efficacy [25]. Perceived stress was assessed with the Perceived Stress Scale consisting of 10 questions, with higher scores indicating a higher level of experienced stress [26]. Total physical activity (hours/week) was measured as the sum of leisure time total physical activity, combined total physical activity during work trips, and total conditioning and functional physical activity.

Socioeconomic factors considered were education (categorized as elementary school, high or vocational school, and college or academic degree), household size (categorized as single, and two or more members), and household gross income (categorized as <€25,000/year, €25,000-64,999/year, and ≥€65,000/year). A currency exchange rate of €1=US $1.14 is applicable.

Factors describing digital abilities were prior use of healthy lifestyle digital apps and internet use several times per day. The factor describing the intervention was the use of the BitHabit app together with face-to-face group coaching.

#### Early User Experience and Use Engagement

User experience was measured after using the app for 1 to 2 months as the rating of the likelihood to recommend the app to others (0-10), which was categorized according to the net promoter score definition [27] into detractors (0-6), passives (7-8), and promoters (9-10).

Furthermore, short-term user engagement was measured as the number of app usage days during the first month of use.

#### User Engagement

Automatically collected log files of BitHabit app use included time-stamped user interactions from which usage metrics, such as usage days, usage sessions and their durations, selected habits, and habit performances, were derived.

#### Outcomes

In this study, 12-month changes in selected T2D risk factors (ie, diet quality measured with the Healthy Diet Index, waist circumference, BMI, and HbA_1c_) were used as outcome measures, which were measured as differences (in absolute scale or in percentage) between baseline and the end of the intervention at 12 months.

### Statistical Analyses

Latent class growth models can be utilized to identify homogeneous subpopulations when studying developmental trajectories from a heterogeneous population [28-30]. This data-driven modeling technique was applied to cluster participants into distinct trajectories of app user engagement. App user engagement was measured as monthly usage days during 2 to 12 months after initiation of app use. This provided 11 repeated measurements on the number of app usage days within 1 month (0-31 days/month). First month usage days were not included in the trajectory analyses but were used as a predictor for trajectory membership in later analyses, as early use is a known predictor of sustained use [31-33]. The number of classes (ie, trajectories) is *a priori* unknown and must be estimated from the data by iteratively fitting alternative models. Models with 1 to 5 classes and varying shapes for the trajectories (linear, quadratic, and cubic) were fitted. Models were estimated with full-information maximum likelihood, and missing data were not imputed, but all available data were used. The method assumes no variation within the trajectories. Unadjusted analyses were conducted. To find the best model, we utilized information from fit indices and the classification performance of the model, as well as clinical interpretation of the trajectories. The Bayesian information criterion was used as a measure of model adequacy, with a lower value indicating a better model with optimal balance between complexity and good fit. The Low-Mendel-Rubin likelihood ratio test with a *P* value <.05 was considered to indicate better fit for an *n*-class model than for an *n*−1 class model. Entropy was used to guide in the classification accuracy of the model, with higher values indicating better classification. Small classes with less than 4% of the total population were not accepted, and to ensure at least 70 participants per group, latent class growth modeling was performed using Mplus Version 8 [34].

Differences in baseline characteristics between the participant groups defined by the identified trajectories were examined with the chi-square test for categorical variables and the Kruskal-Wallis test for continuous variables. Thereafter, a multivariable multinomial logistic regression model was applied to examine the factors associated with the trajectory membership. In a sensitivity analysis, the model was also adjusted for belonging to the DIGI+GROUP. User experience was introduced in the main analysis by conducting a subgroup analysis among the participants who responded to the user experience questionnaire after the first 1 to 2 months of use. Finally, analysis of covariance was used to study associations between the trajectories and short-term changes in T2D risk factors. Models were adjusted for age, sex, and the baseline value of the specific T2D risk factor to account for possible differences in baseline values. Analyses were conducted with IBM SPSS Version 25.0 (IBM Corp) and R Version 4.0.4 (R Core Team). Statistical significance was set at *P*<.05.

### Ethics Statement

The StopDia study was approved by the Research Ethics Committee of the Hospital District of Northern Savo (number: 467/2016). Written informed consent for participation in the study and the use of data from national health care registers was obtained from all study participants.

## Results

### Population Characteristics

**Figure 1:**
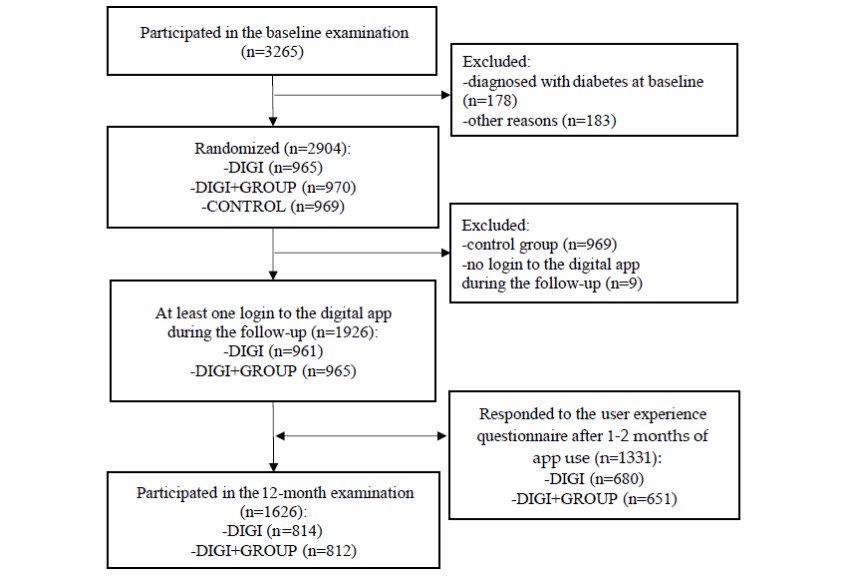
Flow chart of the study population. DIGI, digital intervention group; DIGI+GROUP, group combining the digital intervention and face-to-face group coaching.

The study cohort included 1926 participants in the intervention arms with at least one login to the BitHabit app during the study period (Figure 1). Among them, the mean age was 55.2 (SD 10.0) years, and 79.7% (1535/1926) of the participants were women (Multimedia Appendix 1).

### User Engagement Trajectories

**Figure 2:**
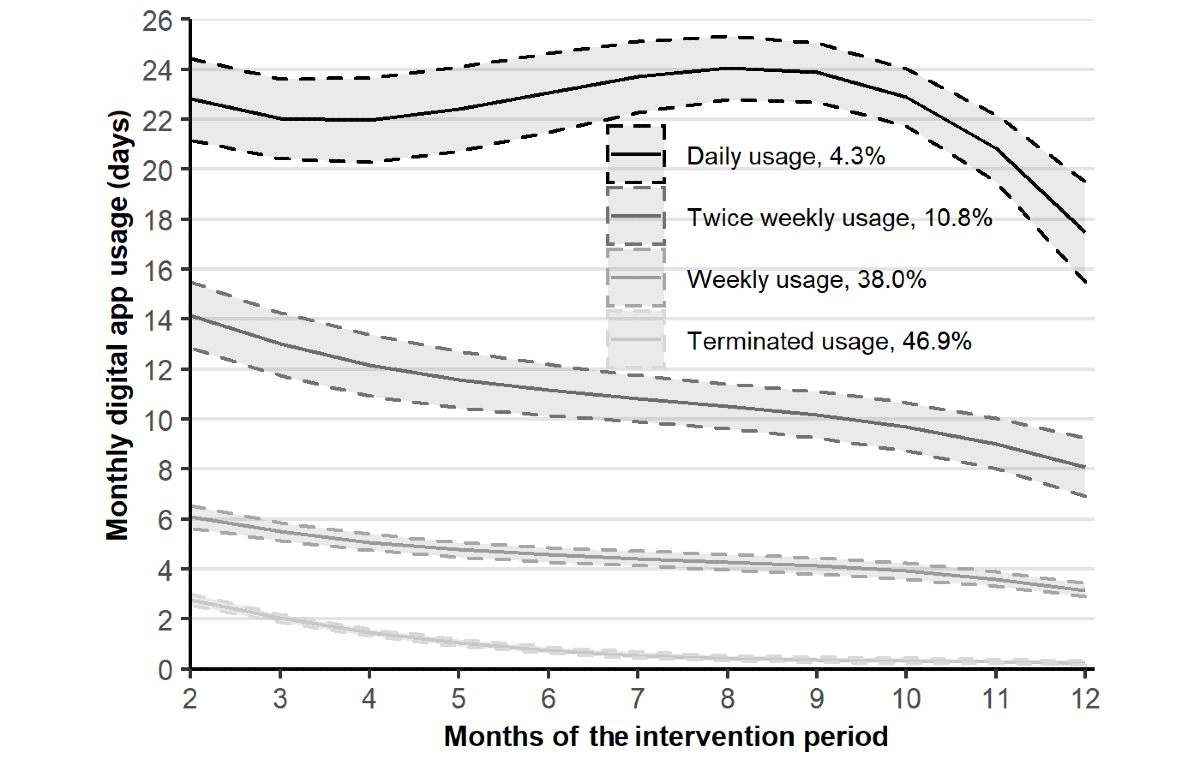
Estimated BitHabit app user engagement trajectories with their 95% CIs.

Based on the Low-Mendel-Rubin likelihood ratio test and the clinical interpretation, a 4-class cubic latent class growth model was identified as the best fitting model with good interpretability (Figure 2; Multimedia Appendix 2). Almost half of the participants (904/1926, 46.9%) had very few usage days in the first months of the study, and they dropped close to zero after 6 to 7 months. Another large group (731/1926, 38.0%) started with approximately 6 usage days per month, which decreased gradually to 3 days per month by the 12th month. A further 10.8% (208/1926) of participants had approximately 14 usage days during the second month with a decrease to 8 days during the 12th month. The remaining 4.3% (83/1926) of participants had almost 23 days of use during the second month, and the usage was sustained with a gradual decrease to 18 days of use during the 12th month.

The identified trajectories were differentiated well when examined with other usage activity metrics (Table 1). Based on the trajectories and all usage metrics, the groups were named as (1) terminated usage, (2) weekly usage, (3) twice weekly usage, and (4) daily usage (Multimedia Appendix 1).

**Table 1:** Distribution of other use activity metrics by trajectories.

Variable	Terminated usage (n=904), median (IQR)	Weekly usage (n=731), median (IQR)	Twice weekly usage (n=208), median (IQR)	Daily usage (n=83), median (IQR)
Number of usage days	12 (5-23)	54 (44-69)	131 (113-157)	262 (228.25-310)
Number of sessions	14 (6-27)	60 (48-77.75)	153 (128-183.50)	310 (257.25-367.25)
Total duration of use (min)	42.73 (19.55-87.18)	153.63 (89.45-263.72)	302.32 (196.24-509.12)	431.15 (288.45-705.86)
Average session duration (min)	3.23 (1.98-5.23)	2.50 (1.42-4.18)	1.97 (1.21-3.44)	1.32 (0.96-2.02)
Usage period from the first day to the last	124 (51.50-245)	356 (343-360)	360 (354-363)	362 (355.25-364)
Weeks in use	9 (4-17)	40 (35-45)	50 (47.5-51)	51 (50-52)
Months in use	4 (2-7)	12 (12-12)	12 (12-12)	12 (12-12)
Number of selected habits	15 (7-26)	32 (20-53)	43.5 (24-61)	50 (33.5-73)
Number of new habits	7 (2-15)	15 (8-28.75)	26 (13-43)	23 (13-46)
Total number of performances	72 (15-269.5)	720 (352-1470.5)	1433 (804-2562.5)	4516 (2405.5-6281)
Number of habits tracked	13 (6-23.5)	28 (17-46)	39 (22-56.5)	49 (31.5-71.75)

### Predictors of User Engagement Trajectories

Active app use during the first month increased the odds of belonging to the more engaged trajectories (odds ratio [OR] 1.65, 95% CI 1.57-1.75 for daily usage; OR 1.45, 95% CI 1.40-1.51 for twice weekly usage; and OR 1.19, 95% CI 1.15-1.22 for weekly usage) than the terminated usage trajectory (Table 2). In addition, older age was associated with increased user engagement compared with the terminated usage category (OR 0.07, 95% CI 0.02-0.19 in those aged <50 years and OR 0.44, 95% CI 0.22-0.87 in those aged 50-59 years for daily usage; OR 0.19, 95% CI 0.11-0.33 in those aged <50 years and OR 0.40, 95% 0.26-0.64 in those aged 50-59 years for twice weekly usage; and OR 0.45, 95% CI 0.34-0.61 in those aged <50 years and OR 0.61, 95% CI 0.47-0.79 in those aged 50-59 years for weekly usage compared with those aged ≥60 years). Women were less likely to be daily users (OR 0.43, 95% CI 0.19-0.98) than terminated users. Better diet quality at baseline, measured with the Healthy Diet Index, increased the odds of belonging to the more engaged trajectories (OR 1.04, 95% CI 1.01-1.08 for daily usage and OR 1.03, 95% CI 1.01-1.05 for twice weekly usage) than the terminated usage trajectory. Prior internet use of several times per day (OR 0.41, 95% CI 0.21-0.79 for daily usage and OR 0.73, 95% CI 0.57-0.95 for weekly usage) decreased the odds of belonging to the more engaged trajectories than the terminated usage trajectory. According to the sensitivity analysis, face-to-face group coaching decreased the odds of belonging to the more engaged trajectories (OR 0.28, 95% CI 0.15-0.52 for daily usage; OR 0.49, 95% CI 0.34-0.73 for twice weekly usage; and OR 0.64, 95% CI 0.52-0.80 for weekly usage) than the terminated usage trajectory (Multimedia Appendix 3). However, there were no more differences between the sexes in trajectory membership.

In a subgroup analysis among 1314 participants who responded to the questionnaire after the first 1 to 2 months of use and had complete data on predictors, app usage days during the first month remained a predictor for trajectory membership (OR 1.56, 95% CI 1.47-1.66 for daily usage; OR 1.37, 95% CI 1.31-1.43 for twice weekly usage; and OR 1.13, 95% CI 1.10-1.17 for weekly usage compared with terminated usage; Multimedia Appendix 4). Furthermore, the detractors classified by the net promoter score had significantly lower odds of belonging to the more engaged trajectories than the terminated usage trajectory (OR 0.23, 95% CI 0.10-0.55 for daily usage; OR 0.19, 95% CI 0.11-0.35 for twice weekly usage; and OR 0.37, 95% CI 0.25-0.55 for weekly usage) compared with promoters of the app.

**Table 2:** Results of multinomial logistic regression analysis.

Variable			Weekly usage^a^ (n=715), aOR^b^ (95% CI)		Twice weekly usage^a^ (n=204), aOR (95% CI)		Daily usage^a^ (n=82), aOR (95% CI)
**Age**							
	<50 years	0.45 (0.34-0.61)^c^		0.19 (0.11-0.33)^c^		0.07 (0.02-0.19)^c^	
	50-59 years	0.61 (0.47-0.79)^c^		0.40 (0.26-0.64)^c^		0.44 (0.22-0.87)^c^	
	≥60 years (reference)	1		1		1	
Women			0.94 (0.72-1.24)		0.73 (0.44-1.21)		0.43 (0.19-0.98)^c^
Obesity			0.87 (0.70-1.08)		0.63 (0.43-0.92)^c^		0.59 (0.32-1.07)
Healthy Diet Index			1.01 (1.00-1.02)^c^		1.03 (1.01-1.05)^c^		1.04 (1.01-1.08)^c^
**Education**							
	Elementary school	0.77 (0.50-1.18)		0.96 (0.48-1.95)		0.68 (0.22-2.07)	
	High or vocational school	1.02 (0.79-1.31)		1.32 (0.85-2.06)		1.33 (0.67-2.63)	
	College or academic degree (reference)	1		1		1	
**Household size**							
	Single	0.92 (0.69-1.23)		0.98 (0.57-1.68)		1.53 (0.69-3.42)	
	≥2 members (reference)	1		1		1	
**Household income per year**							
	≤€24,999	1.45 (0.97-2.16)		1.03 (0.48-2.22)		2.79 (0.86-9.03)	
	€25,000-64,999	1.57 (1.21-2.03)^c^		1.66 (1.05-2.63)^c^		2.87 (1.31-6.32)^c^	
	≥€65,000 (reference)	1		1		1	
Prior use of health lifestyle digital apps			1.04 (0.83-1.30)		1.18 (0.79-1.75)		0.95 (0.50-1.80)
Internet use several times per day			0.73 (0.57-0.95)^c^		0.69 (0.44-1.08)		0.41 (0.21-0.79)^c^
App usage days during the first month			1.19 (1.15-1.22)^c^		1.45 (1.40-1.51)^c^		1.65 (1.57-1.75)^c^

^a^Terminated usage (n=895) as a reference.

^b^aOR: adjusted odds ratio.

^c^*P*<.05.

### Trajectories and Changes in T2D Risk Factors Over 12 Months

**Figure 3:**
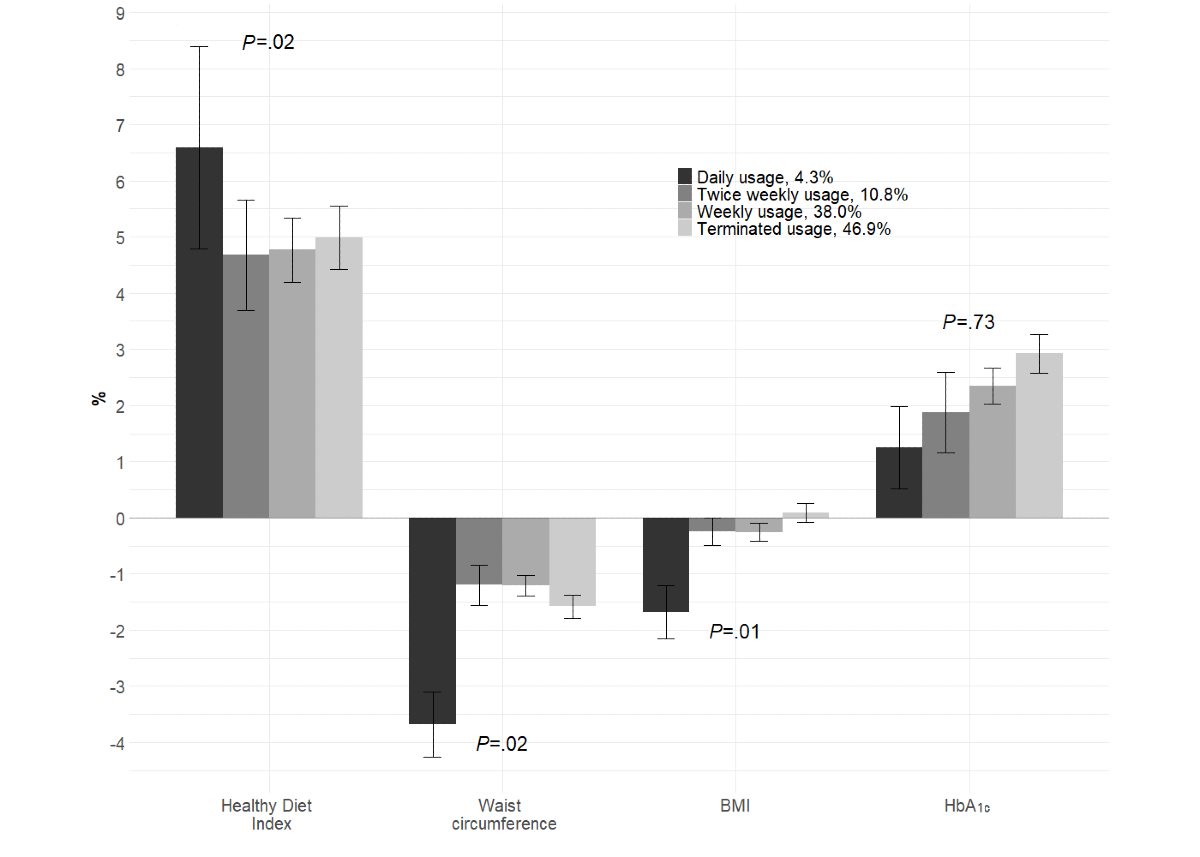
Changes (%) in risk factor levels over 12 months by user engagement trajectories. The error bars represent 95% CIs for means. *P* values are obtained from the analysis of covariance for the main effect of the user engagement trajectory on the change score adjusted for age, sex, and baseline value. HbA_1c_: glycated hemoglobin A_1c_.

High user engagement was associated with 12-month changes in T2D risk factor levels (Figure 3; Multimedia Appendix 5). Compared with the other trajectories, daily usage was associated with a higher increase in the Healthy Diet Index (*P*=.02) and a more pronounced decrease in BMI (*P*=.01) and waist circumference (*P*=.02) at 12 months.

## Discussion

### Principal Findings

Four different trajectories of digital app use were identified with a data-driven technique. We observed that initial user engagement during the first month of the intervention, the net promoter score after the first 1 to 2 months of use, older age, and better diet quality at baseline predicted long-term user engagement. While those who had sustained use once to a couple of times per week showed small beneficial changes in risk factor levels in comparison to those who terminated use, those who used the app almost on a daily basis throughout the study showed the most beneficial changes.

### Comparison With Prior Research

According to our previously reported results, 99% of participants who were allocated to use the BitHabit app logged into the app at least once and selected at least one habit, and 95% reported at least one habit performance during a 6-month follow-up [22]. No significant differences were observed in cumulative usage metrics between the DIGI and DIGI+GROUP, and more than 50% of the participants in both intervention groups continued to use the app at least on a weekly basis in the first 6 months [22]. This study showed that these active users continued to use the app for the entire 12-month intervention. However, according to the sensitivity analysis, receiving face-to-face group coaching in addition to the digital intervention slightly decreased engagement with the app. Participating in the face-to-face coaching may have decreased the need for later digital support. It should be noted that membership in the DIGI+GROUP arm may have mediated the effects in the sensitivity analysis, and therefore, it was omitted from the main analysis.

Overall, BitHabit app use remained remarkably high in comparison with the use of other digital health apps [22]. For example, Goh et al identified 3 short-term 8-week trajectories of weekly use activity for a caloric-monitoring mobile app among T2D patients [35]. A large proportion of the “minimal user” participants (79%) stopped use within the first 2 weeks. Although women seemed to be more active users, we found no gender differences in app use. Older participants were more likely to show frequent use. These background characteristics have been previously reported to be associated with more active use by others as well [36-38]. This result is promising as it demonstrates that digital interventions can be provided to people of all ages. One can only speculate on the reasons for this finding, but a plausible explanation is higher relevance of T2D prevention. Another explanation could be having less work- and family-related daily activities and hence more time for app use. Other than age and household income, no background factors were found to be associated with app use trajectories. However, prior internet use several times per day predicted lower user engagement with the app. Intensive internet use has been associated with lower physical activity in adults [39], which may partly explain the observed association in this study.

App use during the first month and the net promoter score were predictors for user engagement trajectories. Previous studies have shown that short-term treatment adherence predicts future adherence outcomes better than baseline characteristics alone, for example, in the case of adherence to self-management for chronic diseases [31,32]. In a recent study, usage during the first 24 hours after login predicted long-term engagement with a weight loss app [33]. We tested the early adherence hypothesis with a 4-week period, considering it a sufficiently long window of opportunity for monitoring and supporting user engagement in real-world prevention programs.

Theoretically, terminating app use does not necessarily imply disengagement from using the behavior change techniques of the intervention [40]. For example, the habit formation approach in the BitHabit app could have led to termination of app use if participants felt that they had already formed sufficiently strong habits or had learnt to follow the habit formation process without the app. Based on the literature, habit formation takes 10 weeks on average, so even for forming multiple habits, 12 months is a rather long usage time [41]. However, our empirical findings suggest that this was not the case in our study, as those who terminated app use had selected less habits than the other user groups and showed no benefits of the intervention in terms of risk factor levels. Moreover, the benefits increased significantly when the frequency of use increased from weekly to daily. Our results are in line with the findings of previous studies reporting that the effectiveness of digital interventions is dependent on user engagement with the intervention [42-44]. Future studies are needed on whether plausible effects of lifestyle interventions sustain the longest among those with the most active engagement during the intervention or whether effective engagement should be determined through other metrics, such as the content of use (eg, type and quality of habits selected and tracked by the users).

The results indicated that only a minor proportion of the participants, belonging to the trajectory of most active users, achieved marked lifestyle changes. Furthermore, the most active users had better well-being at baseline (ie, better diet, more physical activity, and lower stress levels), which is consistent with previous studies [37,38]. Reaching and engaging those with the highest risks and needs are known challenges in most interventions. Adaptive interventions, that is, monitoring for the early indicators of nonresponse and adapting interventions (eg, augmenting or switching interventions), have been proposed as a solution [45]. This seems especially promising in digital interventions, which inherently enable real-time monitoring of user engagement and detection of waning usage as an early indicator of nonresponse. As this study showed, use activity and user experience after the first month of use predicted long-term user engagement. Thus, early use may be regarded as a window of opportunity where user engagement should be monitored and supported especially in real-world prevention programs. If strategies, such as personalized messages and reminders, fail to re-engage participants with the app, they should be offered alternative interventions, such as face-to-face interventions, or other types of digital solutions for weight management and lifestyle changes [46]. Especially in health care that faces cost containment pressures, this kind of approach would help in providing appropriate types of interventions to different individuals.

### Strengths and Limitations

The strengths of our study include a real-life setting together with a larger sample size and a longer follow-up than in previous studies on the use of digital health apps in persons with prediabetes or T2D [31,43,47]. Additionally, the studied app could be accessed easily with any device as indicated by an almost 100% first login rate, which reduced selection bias and further strengthened our sample.

However, our study also has some limitations. Women were overrepresented in the sample. The proportion of participants who dropped out during the study and, therefore, the proportion of missing data in outcome variables differed between the identified trajectories. We utilized monthly usage days as a measure for user engagement. However, the sensitivity of this measure in detecting changes in risk factors compared with other user activity metrics remains unknown. The composition of selected habits may also be an important factor for risk factor changes and should be examined in future studies.

### Conclusions

Data-driven analysis of digital app user activity is necessary because it reveals true usage patterns. In this study, short-term user engagement, defined as user activity during the first month after the initiation of app use, was found to predict long-term user engagement. Metrics of user experience, based on the net promoter score and measured after early use, was another predictor of long-term user engagement and is a measure that can be easily implemented. These findings could be used in further developments aimed at predicting responses to digital interventions, adapting digital interventions for persons at risk of disengagement, and identifying persons who would benefit the most from using the app.

## Appendix

Multimedia Appendix 1 Baseline characteristics of the study participants in total and by trajectories.

Multimedia Appendix 2 Unadjusted latent class growth analyses for app user engagement (monthly usage days during 2-12 months, n=1926).

Multimedia Appendix 3 Results of a sensitivity analysis conducted with multivariable multinomial logistic regression.

Multimedia Appendix 4 Results of a multivariable multinomial logistic regression analysis among participants in the intervention arms who responded to the user experience questionnaire after the first 1-2 months of use (n=1314).

Multimedia Appendix 5 Mean changes in diabetes risk factor levels between baseline and the 12-month follow-up.

## Abbreviations

DIGI: digital intervention group
DIGI+GROUP: group combining the digital intervention and face-to-face group coaching
FINDRISC: Finnish Diabetes Risk Score
HbA_1c_: glycated hemoglobin A_1c_
OR: odds ratio
RCT: randomized controlled trial
T2D: type 2 diabetes
