# Supplementary material for: Digitally Supported Lifestyle Intervention to Prevent Type 2 Diabetes Through Healthy Habits: Secondary Analysis of Long-Term User Engagement Trajectories in a Randomized Controlled Trial
Source: J Med Internet Res. 2022 Feb 24;24(2):e31530. doi: 10.2196/31530 (PMC8914749; doi:10.2196/31530)
Supplement: Multimedia Appendix 1 [file jmir_v24i2e31530_app1.pdf]

Multimedia Appendix 1. Baseline characteristics of the study participants in total and by trajectories.

	**Total population (n=1926)**	***Terminated usage (n=904)***	***Weekly usage (n=731)***	***Twice weekly usage (n=208)***	***Daily usage (n=83)***	***P* value[a]**
**Clinical variables**						
Women	1535 (79.7)	700 (77.4)	591 (80.8)	174 (83.7)	70 (84.3)	.09
Age, years						<.001
<50	551 (28.6)	332 (36.7)	172 (23.5)	39 (18.8)	8 (9.6)	
50–59	652 (33.9)	328 (36.3)	236 (32.3)	60 (28.8)	28 (33.7)	
≥60	723 (37.5)	244 (27.0)	323 (44.2)	109 (52.4)	47 (56.6)	
BMI (kg/m^{2}), mean (SD)	30.9 (5.4)	31.1 (5.5)	30.9 (5.4)	30.2 (5.1)	30.2 (4.8)	.10
Obesity	1026 (53.3)	507 (56.1)	381 (52.1)	100 (48.1)	38 (45.8)	.06
Missing data	1	1	0	0	0	
Waist circumference (cm), mean (SD)	101.2 (13.2)	101.8 (13.2)	101.2 (13.1)	99.3 (13.8)	100.3 (11.5)	.06
Abdominal obesity	1522 (79.1)	721 (79.9)	580 (79.3)	153 (73.6)	68 (81.9)	.20
Missing data	2	2	0	0	0	
HbA1c (mmol/mol), mean (SD)	36.1 (4.0)	35.6 (4.0)	36.5 (3.9)	36.5 (4.1)	37.3 (4.0)	<.001
Missing data	21	12	6	3	0	
Finnish Diabetes Risk Score, mean (SD)	15.7 (3.5)	15.5 (3.5)	15.8 (3.6)	15.5 (3.6)	16.0 (3.5)	.28
Healthy Diet Index, mean (SD)	62.0 (11.1)	60.4 (11.0)	62.8 (11.0)	65.1 (10.8)	65.7 (11.7)	<.001
Missing data	25	7	14	3	1	
Nutrition Emotional Barriers SelfEfficacy Score, mean (SD)	2.6 (0.6)	2.6 (0.6)	2.7 (0.6)	2.7 (0.6)	2.8 (0.6)	.01
Perceived Stress Scale, mean (SD)	14.9 (6.0)	15.5 (6.1)	14.8 (6.0)	13.8 (5.6)	13.4 (6.2)	<.001
Mean physical activity (SD), hour/week	9.7 (9.0)	8.6 (8.3)	10.5 (9.9)	11.2 (7.9)	11.3 (9.6)	<.001

Missing data	31	14	11	3	3	
**Socio-economic variables**						
Household size						.01
Single	418 (21.7)	180 (19.9)	165 (22.6)	43 (20.8)	30 (36.1)	
≥2 members	1504 (78.3)	723 (80.1)	564 (77.3)	164 (79.2)	53 (63.8)	
Missing data	4	1	2	1	0	
Household income						<.001
≤24,999 €	284 (14.7)	126 (13.9)	111 (15.2)	27 (13.0)	20 (24.1)	
25,000–64,999 €	1024 (53.2)	444 (49.1)	416 (56.9)	117 (56.3)	47 (56.6)	
≥65,000 €	618 (32.1)	334 (36.9)	204 (27.9)	64 (30.8)	16 (19.3)	
Education						.39
Elementary school	146 (7.6)	63 (7.0)	57 (7.8)	19 (9.1)	7 (8.4)	
High or vocational school	524 (27.2)	231 (25.6)	202 (27.6)	63 (30.3)	28 (33.7)	
College or academic degree	1256 (65.2)	610 (67.5)	472 (64.6)	126 (60.6)	48 (57.8)	
**Variables on digital abilities**						
Prior use of health lifestyle digital apps	783 (40.7)	368 (40.7)	295 (40.4)	92 (44.2)	28 (33.7)	.43
Internet use several times per day	1437 (74.6)	715 (79.1)	522 (71.3)	149 (71.6)	51 (61.4)	<.001
**Study-specific variables**						
Intervention group						.02
DIGI	961 (49.9)	418 (46.2)	384 (52.5)	110 (52.9)	49 (59.0)	
DIGI+GROUP	965 (50.1)	486 (53.8)	347 (47.5)	98 (47.1)	34 (41.0)	

Values are frequencies (proportions) unless otherwise stated.
Abbreviations: BMI, body mass index; DIGI, digital intervention; DIGI+GROUP, digital intervention and face-to-face group coaching. [a]*P* values from Kruskal-Wallis test for continuous variables and Chi square test for categorical variables for the differences between trajectory groups.
