# Supplementary material for: Digitally Supported Lifestyle Intervention to Prevent Type 2 Diabetes Through Healthy Habits: Secondary Analysis of Long-Term User Engagement Trajectories in a Randomized Controlled Trial
Source: J Med Internet Res. 2022 Feb 24;24(2):e31530. doi: 10.2196/31530 (PMC8914749; doi:10.2196/31530)
Supplement: Multimedia Appendix 2 [file jmir_v24i2e31530_app2.pdf]

Multimedia Appendix 2. Unadjusted latent class growth analyses for app user engagement (monthly usage days during 2-12 months, n=1926).

**Model**	**Log-likelihood**	**N of param**	**BIC**	**Entropy**	**LMR-LRT**	**Class proportions**				
					P value	*Class 1*	*Class 2*	*Class 3*	*Class 4*	*Class 5*
Linear										
1-class	-66588.4	13	133275.1	NA	NA	100.0				
2-class	-58691.5	16	117503.9	0.994	0.0422	89.7	10.3			
3-class	-55072.9	19	110289.5	0.982	0.0137	77.9	17.1	4.9		
4-class	-53094.5	22	106355.3	0.949	0.1536	47.5	38.2	10.2	4.1	
5-class	-52019.9	25	104228.9	0.961	0.0915	43.9	37.6	11.9	3.9	2.6
Quadratic										
1-class	-72863.9	16	145848.8	NA	NA	100.0				
2-class	-58650.7	18	117437.6	0.994	0.0648	89.7	10.3			
3-class	-54962.8	22	110091.9	0.983	0.0019	78.1	17.1	4.8		
4-class	-52937.4	26	106071.5	0.950	0.0123	47.0	38.1	10.6	4.3	
5-class	-51825.9	30	103878.7	0.962	0.0472	43.8	37.8	11.9	3.9	2.6
Cubic										
1-class	-66573.5	15	133260.4	NA	NA	100.0				
2-class	-58613.8	20	117378.8	0.995	0.0012	89.8	10.2			
3-class	-54903.9	25	109996.8	0.984	0.0003	78.1	17.0	4.8		
4-class	-52855.3	30	105937.6	0.951	0.0365	46.9	38.0	10.8	4.3	
5-class	-51747.6	35	103759.8	0.962	0.1059	43.7	37.7	11.9	4.1	2.6

Abbreviations: BIC, Bayesian information criteria; LMR-LRT, Lo-Mendell-Rubin likelihood ratio test; NA, not available.
