# Supplementary material for: Digitally Supported Lifestyle Intervention to Prevent Type 2 Diabetes Through Healthy Habits: Secondary Analysis of Long-Term User Engagement Trajectories in a Randomized Controlled Trial
Source: J Med Internet Res. 2022 Feb 24;24(2):e31530. doi: 10.2196/31530 (PMC8914749; doi:10.2196/31530)
Supplement: Multimedia Appendix 3 [file jmir_v24i2e31530_app3.pdf]

Multimedia Appendix 3. Results of a sensitivity analysis conducted with multivariable multinomial logistic regression.

	**Weekly usage (n=715)**	**Twice weekly usage (n=204)**	**Daily usage (n=82)**
Age			
<50 years	0.44 (0.33–0.60)	0.19 (0.11–0.33)	0.06 (0.02–0.18)
50–59 years	0.60 (0.46–0.78)	0.40 (0.25–0.63)	0.41 (0.20–0.82)
≥60 years	1 (reference)	1 (reference)	1 (reference)
Women	0.95 (0.73–1.25)	0.74 (0.44–1.23)	0.44 (0.19–1.01)
Obesity	0.87 (0.70–1.07)	0.63 (0.43–0.93)	0.60 (0.33–1.10)
Healthy Diet Index	1.01 (1.00–1.02)	1.03 (1.01–1.05)	1.04 (1.01–1.08)
Education			
Elementary school	0.78 (0.51–1.21)	1.01 (0.49–2.05)	0.80 (0.26–2.47)
High or vocational school	1.01 (0.78–1.30)	1.29 (0.83–2.02)	1.31 (0.66–2.61)
College or academic degree	1 (reference)	1 (reference)	1 (reference)
Household size			
Single	0.93 (0.69–1.24)	0.98 (0.57–1.68)	1.76 (0.78–3.99)
≥2 members	1 (reference)	1 (reference)	1 (reference)
Household income			
≤24,999 €	1.44 (0.97–2.15)	1.00 (0.46–2.17)	2.42 (0.74–7.92)
25,000–64,999 €	1.58 (1.22–2.05)	1.69 (1.06–2.69)	2.85 (1.29–6.30)
≥65,000 €	1 (reference)	1 (reference)	1 (reference)
Prior use of health lifestyle digital apps	1.05 (0.84–1.31)	1.21 (0.81–1.81)	0.98 (0.51–1.88)
Internet use several times per day	0.72 (0.56–0.94)	0.66 (0.42–1.03)	0.39 (0.20–0.76)
DIGI+GROUP	0.64 (0.52–0.80)	0.49 (0.34–0.73)	0.28 (0.15–0.52)

Application usage days during the first month	1.19 (1.16–1.23)	1.47 (1.41–1.53)	1.67 (1.58–1.77)

Adjusted odds ratios (95% confidence intervals) are presented.
Terminated usage (n=895) as a reference.
Abbreviations: DIGI+GROUP, combined digital intervention and face-to-face group coaching.
