# Supplementary material for: Digitally Supported Lifestyle Intervention to Prevent Type 2 Diabetes Through Healthy Habits: Secondary Analysis of Long-Term User Engagement Trajectories in a Randomized Controlled Trial
Source: J Med Internet Res. 2022 Feb 24;24(2):e31530. doi: 10.2196/31530 (PMC8914749; doi:10.2196/31530)
Supplement: Multimedia Appendix 5 [file jmir_v24i2e31530_app5.pdf]

Multimedia Appendix 5. Mean changes in diabetes risk factor levels between baseline and the 12-month follow-up.

**Mean changes in risk factors (SD)**	**Terminated usage (n=812)**	**Weekly usage (n=707)**	**Twice weekly usage (n=199)**	**Daily usage (n=82)**	***P* value[a]**
% change in Healthy Diet Index	4.99 (13.53)	4.77 (14.18)	4.68 (13.48)	6.59 (15.80)	.02
Change in Healthy Diet Index	2.17 (7.64)	2.63 (8.13)	2.49 (7.97)	3.57 (8.32)	.08
Missing data, n (%)	*235 (28.9)*	*97 (13.7)*	*12 (6.0)*	*5 (6.1)*	
% change in waist circumference	-1.47 (5.29)	-1.36 (4.90)	-1.24 (4.64)	-3.23 (5.63)	.02
Change in waist circumference, cm	-1.56 (5.40)	-1.45 (4.89)	-1.33 (4.41)	-3.36 (5.48)	.01
Missing data, n (%)	*149 (18.3)*	*40 (5.7)*	*4 (2.0)*	*4 (4.9)*	
% change in BMI	0.09 (4.44)	-0.26 (4.12)	-0.25 (3.36)	-1.68 (4.25)	.01
Change in BMI, kg/cm^2	-0.00 (1.51)	-0.09 (1.34)	-0.09 (1.00)	-0.51 (1.27)	.03
Missing data, n (%)	*149 (18.3)*	*39 (5.5)*	*3 (1.5)*	*1 (1.2)*	
% change in HbA1c	2.92 (8.56)	2.35 (8.17)	1.87 (9.99)	1.25 (6.53)	.73
Change in HbA1c, mmol/mol	0.92 (3.03)	0.72 (2.97)	0.46 (3.48)	0.34 (2.45)	.49
Missing data, n (%)	*186 (22.9)*	*58 (8.2)*	*5 (2.5)*	*2 (2.4)*	

Abbreviations: BMI, body-mass index. [a]Analysis of covariance for change score adjusted for user engagement trajectory (*P* value), age, gender, and baseline value of the risk factor.
